# Limited knowledge and access to palliative care among women with cervical cancer: an opportunity for integrating oncology and palliative care in Zimbabwe

**DOI:** 10.1186/s12904-020-0523-5

**Published:** 2020-02-13

**Authors:** O. Tapera, A. M. Nyakabau

**Affiliations:** 1 School of Health Systems and Public Health, University of Pretoria, Pretoria, South Africa; 2 Parirenyatwa Group of Hospitals, Radiotherapy Centre, Harare, Zimbabwe

**Keywords:** Cervical cancer, Access, Palliative care, Knowledge, Integration, Sequential mixed methods, Zimbabwe

## Abstract

**Background:**

Cervical cancer is mostly diagnosed at advanced stages among the majority of women in low-income settings, with palliative care being the only feasible form of care. This study was aimed at investigating palliative care knowledge and access among women with cervical cancer in Harare, Zimbabwe.

**Methods:**

Sequential mixed methods design was used, consisting of two surveys and a qualitative inquiry. A census of 134 women diagnosed with cervical cancer who visited two cancer treating health facilities and one palliative care provider in Harare between January and April, 2018 were enrolled in the study. Seventy-eight health workers were also enrolled in a census in the respective facilities for a survey. Validated structured questionnaires in electronic format were used for both surveys. Descriptive statistics were generated from the surveys after conducting univariate analysis using *STATA*. Qualitative study used interview/discussion guides for data collection. Thematic analysis was conducted for qualitative data.

**Results:**

Mean ages of patients and health workers in the surveys were 52 years (SD = 12) and 37 years (SD = 10,respectively. Thirty-two percent of women with cervical cancer reported knowledge of where to seek palliative care. Sixty-eight percent of women with cervical cancer had received treatment, yet only 13% reported receiving palliative care. Few women with cervical cancer associated treatment with pain (13%) and side effects (32%). More women associated cervical cancer with bad smells (81%) and death (84%). Only one of the health workers reported referring patients for palliative care. Seventy-six percent of health workers reported that the majority of patients with cervical cancer sourced their own analgesics from private pharmacies. Qualitative findings revealed a limited or lack of cervical cancer knowledge among nurses especially in primary health care, the existence of stigma among women with cervical cancer and limited implementation of palliative policy.

**Conclusions:**

This study revealed limited knowledge and access to palliative care in a low-income setting due to multi-faceted barriers. These challenges are not unique to the developing world and they present an opportunity for low-income countries to start considering and strategizing the integration of oncology and palliative care models in line with international recommendations.

## Background

Cervical cancer is one of the most prevalent diseases among women in the developing world, with at least 85% of the global reported cases occurring in these contexts [1]. In Zimbabwe, cervical cancer is a growing public health burden and at least 1308 new cases were reported in 2016 [2]. Unfortunately, about 80% of the cervical cancer cases present late in low-income settings when not much can be done to treat the disease. The majority of cervical cancer cases in developing countries are treated with palliative care interventions as curative modalities will not be effective due to advanced disease [3, 4]. However; palliative care interventions in resource-limited settings are scarce or poorly developed hence most women with cervical cancer are at risk of living and dying in severe pain and discomfort [1, 5].

Cervical cancer treatment and care which include modalities such as radical surgery, radiation and chemotherapy as well as palliative care are still largely centralized in Zimbabwe [4, 6]. The costs of treatment are exorbitant and the referral systems are bureaucratic thereby limiting access to diagnosis and treatment among women with cervical cancer who need high level of care. While the government and its partners have been scaling up screening for cervical cancer using visual inspection with acetic acid cervicography (VIAC) and treatment of cervical pre-cancers with cryotherapy and loop electrosurgical excision procedure (LEEP) across the country, diagnosis and treatment of cervical cancer is only available in two tertiary health facilities. The majority of cervical cancer cases are still presented in late stages with palliative care as the only treatment modality [4]. Demand for palliative care services among women with cervical cancer is also growing with increasing burden.

Globally there is evidence of the benefits of integrating oncology and palliative care which includes: improved survival and symptom management, reduced use of futile chemotherapy at the end of life, less patient anxiety and depression, improved patient and family satisfaction and quality of life, and efficiency in the use of healthcare resources [6]. Findings from studies from developed countries which have integrated oncology and palliative care have been mixed. However, the 2018 *Lancet* Commission reported that people in low and middle income settings were living and dying with little or no palliative care. The Commission gave a series of recommendations to quantify serious health-related suffering and proposed an essential package of palliative care which might also be relevant to developed countries. The use of standardized care pathways and multidisciplinary teams to promote integration of oncology and palliative care were proposed by the Commission [6, 7].

Zimbabwe is among the first developing countries to adopt the palliative care approach through Island Hospice, a non-governmental organization (NGO), in 1979. Island Hospice is the centre of excellence for palliative care having trained and built capacity in many countries in Africa including Namibia, Zambia, Botswana, Kenya, and South Africa. For many years the organization attended to most oncology patients before it transitioned in 1987 to supporting patients with HIV/AIDS and others with chronic diseases. In the 2000s Island Hospice extended the philosophy, knowledge and skills of the hospice model through capacity building of communities and health care professionals in the public health institutions to improve awareness and support the integration of palliative care into the routine health delivery system [8]. In 2014 Zimbabwe rolled out the national palliative care policy which reported that there was a huge need for palliative care interventions for people suffering from HIV and AIDS, cancer and other chronic diseases [9]. Recently, the Ministry of Health and Child Care and its partners developed a national palliative care strategy framework; however the policy is yet to be fully implemented due to limited resources and competing priorities. The country also has inadequate palliative care trained personnel in the health facilities. Since the launch of the strategic framework, 315 health workers were trained in palliative care though this is still a small number considering the growing needs. Palliative care service coverage remains limited and most health facilities experience frequent stock outs of palliative care medicines across the country. Island Hospice has been supplying the majority of opiods in public health facilities, however, access to these medicines still remains limited in the country and reliance on donor funding is unsustainable [10].

World Health Organization defined palliative care as “an approach that improves the quality of life of patients (adults and children) and their families who are facing problems associated with life-threatening illness. It prevents and relieves suffering through the early identification, correct assessment and treatment of pain and other problems, whether physical, psychosocial or spiritual” [11]. In the Zimbabwean context palliative care concept is largely relegated to terminal palliative care and a myriad of misconceptions are associated with the approach [8]. Anecdotal evidence in Zimbabwe has shown that the approach is stigmatized in communities and it is associated with death. Furthermore, the approach is still not yet fully integrated into the routine care and is still largely supported by NGOs. Understanding of palliative care and its integration into the health system remains limited in Zimbabwe despite the long history of using this approach since 1979. With the growing burden of cancers, notably cervical cancer, palliative care has become a crucial intervention for patients and families [6, 8]. Recently, the government with the support of Island Hospice and WHO begun the process of piloting the integration of palliative care and oncology in line with international best practices, with the hope of scaling up in future [12]. In this paper we report on our study that aimed at understanding palliative care knowledge and access among women in Zimbabwe with cervical cancer to provide evidence that could be used in programme and policy improvement.

## Methods

### Study design

A sequential explanatory mixed methods design was used for this study, with descriptive cross-sectional surveys being the major study and qualitative inquiry being a minor study. The purpose of the qualitative inquiry was to understand deeper issues, and to seek to explain surprising and unexpected results from the surveys. Two cross-sectional surveys namely: patient and health worker surveys were conducted on randomly selected participants in two cancer treating health facilities and one palliative care provider in Harare, Zimbabwe. The health facilities selected were Harare Central Hospital and Parirenyatwa Group of Hospital, and the palliative care provider was Island Hospice. Selection of health facilities was based on the provision of cervical cancer treatment and palliative care which is predominantly provided by Island Hospice.

### Target population

The target population for the study consisted of women with cervical cancer, men, caregivers, health workers and stakeholders working in the cancer spaces in Zimbabwe. Women with cervical cancer who were at least 25 years of age were considered for enrolment in the survey. The inclusion of healthy women in qualitative inquiry was aimed at fully understanding their knowledge and perceptions about cervical cancer palliative care to inform future programmes and policies. Men and caregivers were included in the qualitative phase of the study as they play a crucial role in access of health care services [13, 14].

### Sample sizes and participant selection

**Fig. 1:**
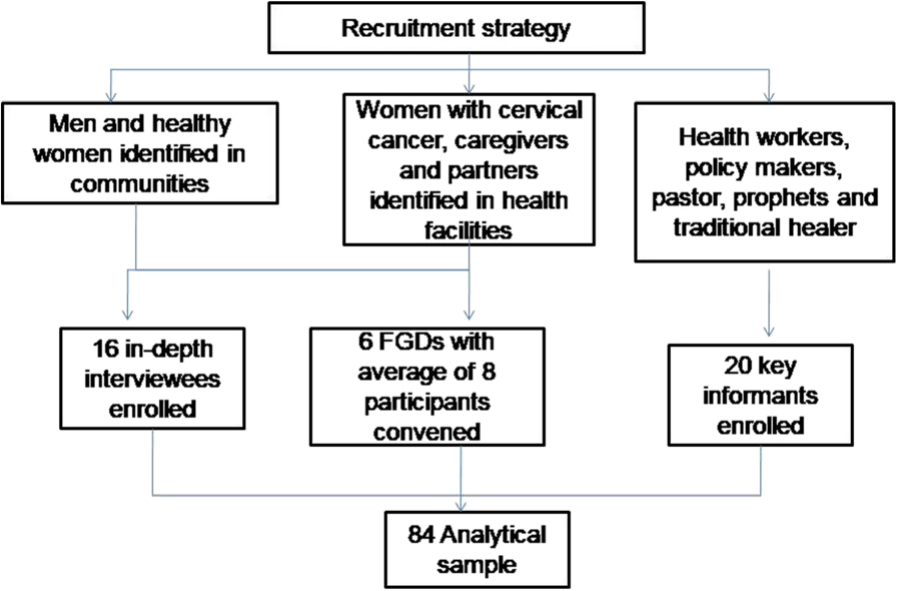
Flow diagram of qualitative study recruitment

For the patient survey, a census of women with cervical cancer who visited the three selected health facilities was conducted between January and April 2018. A total sample of 134 women with cervical cancer was achieved. In the health worker survey a total of 78 health workers working in cervical cancer screening, treatment and palliative care sections/departments in the selected health facilities were enrolled using a census approach. The sample sizes for the qualitative data collection methods were guided by the saturation principle [15]. A total of 84 participants were selected purposively for the qualitative inquiry. The sample sizes achieved in in-depth interviews, FGDs and key informant interviews were 16, 48 and 20 respectively. The 48 participants selected for focus group discussions (FGD) were organized into 6 groups which had an average number of 8 people per group (see Fig. 1). Focus group and in-depth interview participants were selected during patient survey based on their knowledge of the disease, having good or bad experiences in seeking screening, diagnostic and treatment services and to achieve a diversity of perspectives with regards to the issues under investigation. A diverse group of young, middle aged and old patients who resided in rural and urban areas and with different disease stages were selected for both in-depth interviews and FGDs. Key informants were selected based on their experience in interacting with cervical cancer patients. These included health workers, policy makers, community leaders (pastors, prophets and traditional healers) and programme managers in NGOs providing cervical cancer services in Zimbabwe and snowball sampling technique was used to select the participants [16].

### Data collection

In the two surveys, validated structured questionnaires [17] programmed in *SuveytoGo* software [Dooblo, Israel] in an android tablet were used to collect data from face-to-face interviews with participants [15]. Electronic data collection enabled real-time uploading of data to a cloud server and quality of control data while fieldwork was still on-going. Qualitative data collection tools which consisted of in-depth interview, key informant and FGD guides were finalized with input from survey data analysis. Qualitative data were collected using audio-recorder and notebooks. All participants consented in writing before interviews/discussions and either English or Shona language was used depending on the preference of the participant(s). Participants interviewed or enrolled for FGDs in health facilities or in communities far away from their residences were provided with an average of US$ to cover their transport expenses.

### Data analysis

Data from the surveys was downloaded from a cloud server in *Excel* format which was then imported into STATA. After data cleaning, univariate analyses were conducted to yield descriptive statistics, using as outcome variables cervical cancer palliative care knowledge and access to palliative care among women with the disease. All survey analyses were conducted independently using *STATA* version 14 software (StataCorp, Texas). For the qualitative inquiry, all recordings were transcribed and translated into English verbatim. The transcripts were loaded into *Dedoose* software where line-by-line coding was conducted using a thematic coding framework that had been developed from literature and survey findings. After coding, analysis was conducted using the same software to identify the salient emerging themes relevant to this study from the data. The main themes were reported and supported by direct quotes from some of the participants.

## Results

### Quantitative results

#### Characteristics of participants

A total of 212 participants responded to the two surveys, 134 women with cervical cancer and 78 health workers. The mean ages of participants in the patient and health worker surveys were 52 years (SD = 12) and 37 years (SD = 10), respectively. Table 1 shows the characteristics of women with cervical cancer: 94% had advanced disease (FIGO stage ≥2b2), 61% had at least secondary education, 53% had no income, 67% were unemployed, 56% were poor or in middle class and 60% were widowed, divorced or separated. Sixty-nine percent (69%) of women with cervical cancer had received treatment yet only 13% reported receiving palliative care.

**Table 1:** Socio-demographic characteristics of women with cervical cancer in Harare, Zimbabwe

Participant type	Women with cervical cancer
**Socio-demographic variables**	**[*N*** **= 134] (%)**
**Residence**	
Urban	74 (55)
Urban_Low density	3(2)
Urban_High density	67(50)
Urban_Medium density	4(3)
Rural	60(45)
**Age (years)**	**Mean (52)**
25–34	6(4)
35–44	31(23)
45–54	41(31)
55 or more	56(42)
**Ethnicity**	
Shona	130(97)
Ndebele	2(1)
Other	2(2)
**Marital status**	
Married/co-habiting	52(39)
Never married	1(1)
Widowed	59(44)
Divorced or separated	22(16)
**Religion**	
Roman Catholic	34(25)
Protestant	24(18)
Pentecostal	34(25)
Apostolic sect	34(25)
Other	8(7)
**Education**	
Primary	43(32)
Secondary	75(56)
Higher	6(5)
None	10(7)
**Household head education**	
Primary	16(12)
Secondary	50(37)
Higher	14(10)
Not Applicable	5(4)
None	49(37)
**Occupation**	
Unemployed	90(67)
Student	3(2)
Professional	3(2)
Police/Military/Security	12(9)
Trucker/transport business	1(1)
General worker	1(1)
Self employed	5(4)
Vendor	16(12)
**Occupation of household head**	
Unemployed	25(19)
Farm worker	2(1)
Professional	23(17)
Police/Military/Security	5(4)
Trucker/transport business	1(1)
General worker	0
Self employed	30(22)
Vendor	1(1)
Other	47(35)
**Personal income (US$)**	
No income	77(57)
< 200	32(24)
200–400	19(14)
430 or more	6(4)
**Household income (US$)**	
No income	71(53)
< 600	53(40)
600–1000	6(4)
1200 or more	4(3)
**Medical insurance/aid**	
Yes	27(20)
No	107(80)
**Wealth quintiles**	
Poorest	7(5)
Poorer	32(24)
Middle	36(27)
Richer	26(19)
Richest	33(25)
**Sources of cervical cancer information**	
Radio	31 (25)
TV	27 (21)
Health workers	57 (45)
Other	11 (9)
**Knowledge that cervical cancer is treatable**	
Yes	113 (84)
No	7 (5)
Don’t	14 (11)
**Cervical cancer disease stages**	
≤ 2b1	6 (4)
2b11–4	128(96)
**Cervical Cancer treatment status**	
Yes	92(69)
No	42(31)
**Received palliative care**	
Yes	17(13)
No	117(87)

Table 2 shows low knowledge (32%) of where to seek for palliative care services. Fewer women with cervical cancer associated *treatment* with pain (13%) and side effects (32%). However, more women associated cervical cancer with bad smells (81%) and death (84%).

**Table 2:** Knowledge and perceptions of cervical cancer and palliative care among women with cervical cancer in Harare, Zimbabwe

Variable	Women with cervical cancer participants [*N* = 134] (%)
Knowledge of where to seek for palliative care services	43 (32)
Local health facilities offer counseling to cervical patients and their families	111 (83)
Local health facility offers health education about cervical cancer to women.	92 (69)
Cervical cancer treatment is painful	18 (13)
Cervical cancer treatment has side effects	43 (32)
Cervical cancer is painful	33 (25)
Cervical cancer is smelly	109 (81)
Cervical cancer can cause death	113 (84)

Table 3 shows that a high proportion (72%) of health workers had received adequate training to provide *treatment* and palliative care of cervical cancer patients. Seventy-six (76%) of health workers reported having guidelines for the *treatment* and palliation of cervical cancer patients in their health facilities. Most health workers (96%) reported that the majority of women with cancer of cervix presented late. Only one health worker referred patients for palliative care at Island Hospice or other service providers or to a palliative care specialist three months prior to the survey. Less than half of health workers reported that most patients with cervical cancer were accessing *treatment* and palliative care and this was based on their professional experiences with cervical cancer patients/survivors. Stock-outs of analgesics across the three-step ladder three months prior to the surveys were reported by 22% of the health workers. Half of the health workers perceived that their facilities had adequate analgesics for palliative care of patients with cervical cancer. A large proportion of health workers (76%) reported that the majority of patients were responsible for purchasing their own analgesics for palliative care.

**Table 3:** Knowledge and perceptions of palliative care among health workers

Variable	Health worker participants [*N* = 78] (%)
Adequacy of training to provide cervical cancer treatment and palliative care	56 (72)
Availability of clinical guidelines for cervical cancer treatment and palliative care.	59 (76)
Most women present late with cervical cancer	75 (96)
Referral for palliative care	1 (1)
Most women having access to treatment and palliative care for cervical cancer	37 (47)
Stock-outs of analgesics in previous 3 months	17 (22)
Adequacy of analgesics for palliative care	39 (50)
Patients buy their own analgesics for palliative care	59 (76)

### Qualitative results

#### Main theme: palliative care knowledge and access is limited

The salient main theme from the qualitative inquiry was that among patients and health workers alike palliative care knowledge is still limited in Zimbabwe. Palliative care access or perceptions of it were reported as limited even among women who had access to other *treatment* modalities.

#### Subtheme: poor understanding of palliative care

One of the major subtheme emerging from this study was that there is a general perception that palliative care is end-of-life care and should only be given when all other interventions have failed and the patient is about to die.

One FGD healthy woman participant from Hopely community described palliative care as follows:

*“If the doctors tell you that the condition can no longer be treated we should be able to comfort our patient and to have a peace of mind. The hospital staff told us that there was no help they could offer to our patient unless if we had come earlier and she was given pain killers though she could not survive for long. Generally, advanced cervical cancer is seen as a death sentence in our communities*”

*“You walk into a rural health facility and you ask nurses about cervical cancer or cancer in general but they have no clue of what it is but we are asking people in the community to go to the clinic where the nurses don’t know anything about cancer.”-*Cancer Programme Manager

One pharmacologist reported the need for strengthening health education:

*“I think people need to be educated, and we need to inform people why it helps, and how long they will survive getting this help. Some would have seen people with cervical cancer suffering and they would just think they are dying”.*

#### Subtheme: stigmatization of cervical cancer and palliative care

Some participants reported that cervical cancer and palliative care were associated with stigma and people linked them with the nearing of death. One informant said:

*“I remember one of my patients who was being seen by Island Hospice and they used to have cars with the Island Hospice logo and I mean if you are staying in high density areas you are like identified like, ooh those ones that see that woman who is dying from cervical cancer and so the patient associated attending palliative care services as something that is associated with dying such that they would rather shun those services because then by accepting services from them means they are accepting that they are dying and that’s one of the problems that palliative care services have”.*

Another health professional informant also described advanced cervical cancer and palliative care as associated with stigma.

*“Maybe they stigmatize the disease [cervical cancer] and palliative care approach because nothing else can be done, which is true because when you offering palliative care you are just offering symptomatic care either pain relief or wound care. So I think communities need better understanding of the disease [cervical cancer] itself and to know that nothing may be done to cure advanced disease and all that needs to be done is just to let that person live a more comfortable life until the end”-* Senior Gynaecologist

#### Subtheme: palliative care is relegated to hospices or nursing homes by health workers

There is a general perception among some health workers that palliative care is only provided by hospices and is the last level of care for advanced disease. This perception has also permeated to the patients who believe that when doctors can longer do anything that is when they need palliative care.

*“Yes, but when it comes to cancer the Island Hospice, the Cancer Centre are the two names linked to palliative care and patients would want to know if the doctors have got nothing to do for them and theirs is a hopeless situation”. –* Senior Radiographer

#### Subtheme: pain management is compromised by high costs of medicines

High costs of medicines for cancer patients are a barrier to accessing pain management which is a key component of palliative care. While some organizations like Island Hospice provide it for free, because of the high costs of treatment of cancer some patients just give up seeking or pursuing health services.

*“…..for some time there has been a cost attached to it and that cost has been a deterrent”. -* WHO expert

*“……if you are suspected of cervical cancer the next stages needed for confirmation are very expensive….”-* Hopely FGD participant, healthy woman.

*“…..in Zimbabwe drugs aren’t available if they are there they are expensive and that’s another barrier to access.”-* Pharmacologist

#### Subtheme: limited implementation of palliative care policy framework

One of the emerging subthemes from key informants was that the existing palliative care strategy framework was not fully implemented in the country due to limited resources. Some key informants had the following to say:

*“In terms of palliative care when we think of palliative care specialists it’s not really incorporated… I don’t know at what stage the patient has to get to be before a palliative care specialist but I don’t think this is happening there.”-* Pharmacologist

*“……..so that’s why I am saying we don’t have that system of palliative care on the government side.”-* Senior Gynaecologist

*“…..health system here we don’t even believe that it can handle the number of clients that this country has, you know how many centres we have, how many oncologists do we have and so forth”-* WHO expert

*“…….we need to have guidelines that are set up by a multi-disciplinary team. It must be the whole team. It must be pathologists, it must be oncologists, it must be gynaecologists, it must be the nurses, epidemiologists coming together looking at the data, looking at the population coming up with guidelines that work for us here in Zimbabwe. At the moment there are no guidelines everybody is just doing what they think. You know we have MSF they are doing their own project, Family health care their own….”-* Senior Pathologist

## Discussion

This study revealed a number of issues with regards to palliative care for cervical cancer. There is limited knowledge of palliative care approach based on the low proportions of women with cervical cancer who knew where to go to seek for palliative care. Few women associated cervical cancer treatment as causing with pain and side effects. The majority of women with cervical cancer reported the availability of counseling services and health education for cervical cancer in their local health facilities. The majority of women associated cervical cancer with pain, bad smells and death. A high proportion (69%) had received treatment, however only 13% reported receiving palliative care. Most health workers reported having knowledge about guidelines for treatment and palliation of cervical cancer patients and receiving adequate training for their roles in treatment and palliation of cancer patients. However, only one health worker reported referring cervical cancer patients for palliative care services at Island Hospice or other providers. Most cervical cancer patients sourced their own analgesics from private pharmacies. Qualitative findings revealed: limited or lack of cervical cancer knowledge among nurses especially in primary health care, the existence of stigma among women with cervical cancer and limited implementation of palliative strategy framework.

The findings of this study showed limited knowledge or poor understanding about palliative care for women with advanced cervical cancer. The *Lancet* Commission recently reported that public awareness of palliative care and associated services were scarce even in developed countries [6]. In a UK study, only 19% of patients recognized the term palliative care while 68% understood the role of hospices and 67% understood the roles of specialist palliative care nurses [18]. Hirai and colleagues [19] reported in their study that 63.1% of respondents had no knowledge of palliative care and as little as 0.5% were using palliative care services in Japan. These findings are consistent with those from our study. Our qualitative work revealed that poor understanding of palliative care could be driven by stigma associated with advanced cervical cancer, palliative care and the fact that it affects the cervix, a private part of the body of a woman [6]. Other studies have also supported this finding that stigma surrounding cancer presented barriers to treatment and care. The social, emotional and financial devastation that accompanies the diagnosis of cancer is driven by cultural myths and taboos surrounding the disease [6, 20–22].

Low perceptions of pain and side effects associated with cervical cancer treatment could be explained by the fact that most women have the perception that cervical cancer is painful. Therefore, any pain or side effect experienced during or after treatment is negligible or is attributed to the disease and not to the treatment modalities themselves. Furthermore, survival outcomes for women treated in developing countries are low [1, 20] hence there may not be much knowledge of long term side effects. This is supported by the general perception from our qualitative inquiry that advanced cervical cancer is a *“death sentence*” hence if one has the disease they will not live long. Awareness and health education in the communities including among women with cervical cancer and health workers require further strengthening to improve knowledge about palliative care approach.

Limited counseling and health education services in local health facilities were reported in this study. Qualitative data also supported this finding which revealed limited or lack of knowledge about cervical cancer among nurses particularly in rural areas. This further suggests limited policy framework implementation and absence of priorities for palliative care from the government of Zimbabwe. There is a common misconception that palliative care is end-of life care only and coupled with stigmatization of death and dying most people have not embraced the contemporary concept of palliative care which starts at diagnosis. This has resulted in people resorting to “palliative care” interventions as the last resort and this is also common even among health workers [6]. Studies in developed settings have also reported that patients with advanced diseases were referred to hospices or nursing homes when they could not stay in their homes. However, other studies have shown that the quality of life of patients who are kept in hospices or nursing homes is lower than those who stay with their families at home [6, 21–23]. While the government of Zimbabwe has rolled out the palliative strategy framework, more resources and priorization are required to fully implement the policy.

Access to palliative care was limited even among patients who had received some other forms of treatment in this study. Only one health worker reported referring cervical cancer patient for palliative care. Greer and colleagues in their USA study reported that late referral to hospices deprives the entire family from comprehensive support care they could beneficial from [24]. Qualitative findings supported the results from the surveys, suggesting that patients were referred only when all other interventions had “failed” or as the last resort or when the patient is nearing death. There are also no clear guidelines about the pathways of care in health facilities across the country. While a high proportion of health workers reported availability of clinical guidelines for the *treatment* of cervical cancer including palliative care, qualitative data revealed that the guidelines were not updated, standardized and fully implemented in treating health facilities. This status quo suggests limited implementation of the national palliative care policy on the backdrop of limited resources and competing priorities in the health sector [10]. High costs of medicines and other *treatment* procedures were reported as barriers to accessing pain management in our context. These barriers were also reported in the *Lancet* Commission [6] and other studies [25–27]. There are opportunities for Zimbabwe to engage the private sector, local and international partners to support the full integration of palliative care into the health system for optimal outcomes.

## Limitations and strength of the study

The main limitations of this study were: findings from cross sectional surveys may not be used to infer causality and results from qualitative inquiries may not be generalizable beyond the study settings. However, the study had its own strength; the use of both quantitative and qualitative methods provided better research outcomes for policy recommendations.

## Conclusion

In conclusion, our study revealed limitations in knowledge and access to palliative care due to a myriad of multi-faceted barriers in a low-income context. Furthermore, these challenges are not unique to the developing world and they present an opportunity for low-income countries to start considering and strategizing the integration of oncology and palliative care models in line with international recommendations. Low-income countries could rally behind developed countries that are in advanced stages of integration using different models informed by evidence and their contextual circumstances. Sound policies, strong political will and significant investments underpin the success of any integration model, thus collaborations with industry, academic institutions and international organizations are recommended.

## Data Availability

The datasets used and/or analyzed during the current study are available from the corresponding author on reasonable request.

## Abbreviations

FGD: Focus Group Discussion
VIAC: Visual inspection with acetic acid cervicography
WHO: World Health Organization
